# Investigation on the Removal Performances of Heavy Metal Copper (II) Ions from Aqueous Solutions Using Hydrate-Based Method

**DOI:** 10.3390/molecules28020469

**Published:** 2023-01-04

**Authors:** Xiaobing Lan, Jun Chen, Yang Xie, Fenglong Hu, Changzhong Chen, Dongdong Li, Jianhong Jiang, Bin Deng

**Affiliations:** Hunan Provincial Key Laboratory of Xiangnan Rare-Precious Metals Compounds Research and Application, School of Chemistry and Environmental Science, Xiangnan University, Chenzhou 423000, China

**Keywords:** heavy metal ions, copper (II) ions, removal, wastewater, hydrate

## Abstract

Since heavy metal ion-contaminated water pollutionis becoming a serious threat to human and aquatic lives, new methods for highly efficient removal of heavy metal ions from wastewater are important to tackle environmental problems and sustainable development. In this work, we investigate the removal performances of heavy metal copper (II) ions from aqueous solutions using a gas hydrate-based method. Efficient removal of heavy metal copper (II) ions from wastewater via a methane hydrate process was demonstrated. The influence of the temperature, hydration time, copper (II) ions concentration, and stirring rate on the removal of heavy metal copper (II) ions were evaluated. The results suggested that a maximum of 75.8% copper (II) ions were removed from aqueous solution and obtained melted water with 70.6% yield with a temperature of −2 °C, stirring speed 800 r/min, and hydration time of 4 h with aninitial copper concentration of 100 mg/L. The initial concentration of copper (II) ions in the aqueous solution could be increased to between 100 and 500 mg/L. Meanwhile, our study also indicated that 65.6% copper (II) ions were removed from aqueous solution and the yield of melted water with 56.7%, even with the initial copper concentration of 500 mg/L. This research work demonstrates great potential for general applicability to heavy metal ion-contaminated wastewater treatment and provides a reference for the application of the gas hydrate method in separation.

## 1. Introduction

Rapid population growth and economic development has created a growing demand for consumer goods, which has also led to a serious rise in various pollution problems [1,2,3,4,5]. Along with a plethora of pollution issues, water pollution has become a key challenge for sustainable development. It is well known that water is indispensable to humans in all aspects of human life and the available water resources on the earth are quite limited. Currently, the demand for agricultural, medical and industrial activities has significantly increased. This has led to various hazardous pollutants into water resources and has resulted in serious pollution of surface and ground water resources. Typical water pollutants such as microplastics, heavy metals, bacteria, viruses, and radiological contaminants are known to have seriously affected the usage of water resources [6,7,8]. Among them, hazardous heavy metal pollution of wastewater is one of the utmost significant environmental problems and endangers human beings throughout the world [9,10,11,12]. Although heavy metals can generally be detected in traces, they are still hazardous. Heavy metals are highly toxic and stable in aquatic environments and do not degrade. They can build up in significant amounts in the human body once they reach the food chain and cause serious health issues when metals are consumed beyond the allowed concentration limit [13,14,15]. On the other hand, a recent report demonstrated that heavy metals contribute to the heterogeneity of water environments via metal–algae interaction. Heavy metals affect algal communities, which could cause a potential risk of algal blooms. Harmful algal blooms seriously affect ecological environments because harmful cyanobacteria occupy the habitat of other organisms, resulting in biological community structure and ecological balance in aquatic environments becoming disrupted and biodiversity decreasing [16,17]. Thus, efficient decontamination of heavy metals from water is an urgent and challenging issue.

To this end, a variety of effective methods have been developed to remove heavy metal ions from water, such as chemical precipitation, ion exchange, the oxidation–reduction method, membrane separation, electrochemical and adsorption [18,19,20,21]. However, these methods often cause some side effects and include excessive pretreatment steps. For example, chemical precipitation can produce large amount of sludge that can only be treated with great difficulty, or ineffectively when metal ion concentration is very low, while flotation and electrochemical technology involve high initial investment and operation costs [22,23]. Some new methods, such as electro-adsorption or photocatalytic reduction of heavy metals, are also reported in very recent years [22,24]. However, it is still highly desired to design a noveland efficient means for removal of heavy metals for the sustainable development of the environment.

**Figure 1:**
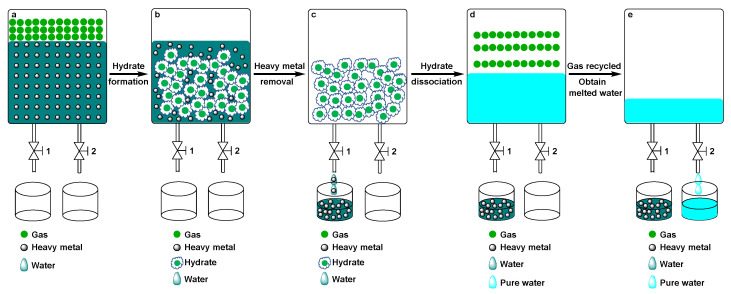
Schematic diagram of heavy metal separation from aqueous solution. (**a**) Before of hydrate formation; (**b**) Hydrate formation; (**c**) Heavy metal removal; (**d**) Hydrate dissociation; (**e**) Gas recycled and obtain melted water.

Recently, clathrate hydrate-based technologies have attracted considerable attention for mixture separation, such as gas mixture separation, water desalination, and purification, which have the advantages of ease of operation, low toxicity and good selectivity [25,26,27,28]. Clathrate hydrates are ice-like substances consisting of host water molecules which are hydrogen-bonded to form cages that encapsulate different guest molecules [29,30]. The basis for hydrate-based gas separation is guest compound selectivity, which is one of the unique properties of hydrates. Gas hydrate is formed by gas molecules and water at a low temperature and under high pressure [31,32,33]. In theory, heavy metal ions could also be separated by being repelled out of the hydrate phase from the wastewater when host water molecules with gas guest molecules form hydrate. A detailed process for a gas hydrate-based method for heavy metal removal is shown in Figure 1. Under a certain temperature and pressure, pure water crystallizes as a solid hydrate phase from aqueous solution with the help of a gas molecule and all the metal ions are ejected into the effluent during the formation of hydrate (Figure 1a,b). As a result, only pure water and gas are left in the solid hydrate. The residual water with metal ions was thrown away, leaving significant solid hydrate (Figure 1c). The solid hydrate was further dissociated via thermal stimulation or depressurization to yield pure water and the gas could be further recycled (Figure 1d,e). In this context, gas hydrate-based technology has recently emerged as a promising option for the removal of heavy metal ions from water [34,35,36,37]. For example, Zhao and their co-works first performed heavy metal separation experiments from aqueous solutions using a hydrate-based method [38]. In our previous work, we have also measured the removal efficiencies of heavy metal chromium and nickel form their aqueous solutions using a hydrate-based method [39,40].

As we know, copper is one of the most common heavy metals. Excessive copper can harm the nervous system and organs, especially the liver and gallbladder, leading to neuritis and cirrhosis. Therefore, removing copper ions from wastewater is a very important project. In this work, we have performed heavy metal copper (II) ion separation experiments from aqueous solutions using a gas hydrate-based method. This method has several advantages: (a) mild operating conditions and cooling energy is saved, (b) the process is simple, and no raw material is lost in theory, (c) it is safe to handle and cost effective. The obtained data were also analyzed to evaluate the possibility of heavy metal copper (II) ion separation using a gas hydrate-based method.

## 2. Results and Discussion

### 2.1. Effect of Temperature on the Copper (II) Ions Removal Efficiency

**Figure 2:**
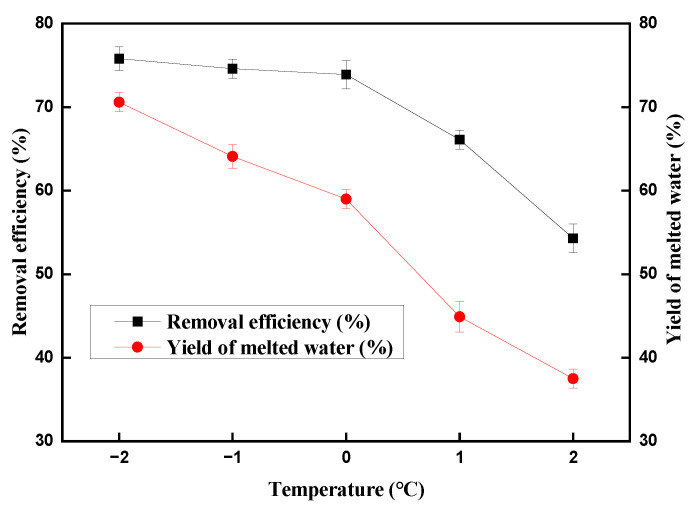
Effect of temperature on the removal of copper (II) ions and the yield of melted water.

To begin our study, we use CuSO_4_ as copper (II) ion precursors and model solutions were prepared with concentrations in 100 mg/L, under conditions of a stirring speed of 800 r/min and a hydration time of 4 h. Methane hydrate was formed under 6.0 MPa pressure and the experimental temperatures were −2 °C, −1 °C, 0 °C, 1 °C and 2 °C respectively. As shown in Figure 2, a series of experiments was carried out to evaluate the experimental temperatures. When the temperature was −2 °C, copper (II) ion removal efficiency was 75.8% and the yield of melted water was 70.6%. Although the copper (II) ion removal efficiency was 74.6% and 73.9% respectively when the temperature increased to −1 °C and 0 °C, the yield of melted water sharply decreased to 64.1% and 59.0%, respectively. When the temperature increased to 1 °C and 2 °C, the copper (II) ion removal efficiency and the yield of melted water both sharply decreased. Thus, we use −2 °C as the temperature for the following experiments.

### 2.2. Effect of Hydration-Time on the Copper (II) Ion Removal Efficiency

**Figure 3:**
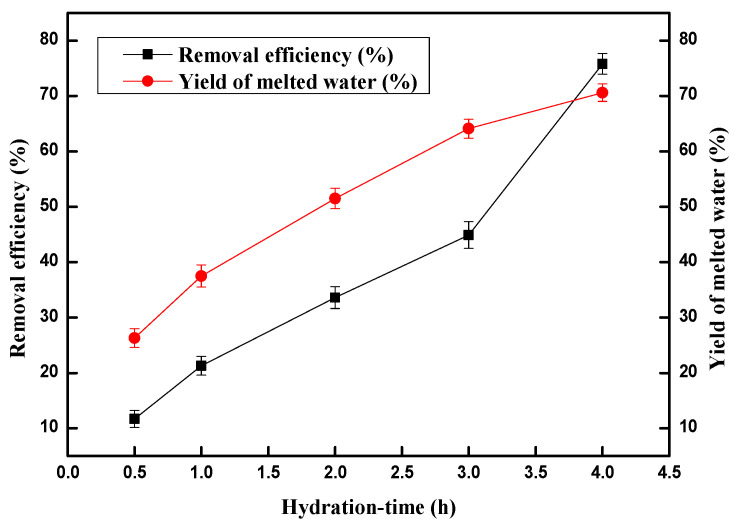
Effect of hydration time on the removal of copper (II) ions and the yield of melted water.

After the optimal temperature −2 °C was determined, we began to explore the effect of hydration time on the copper (II) ions removal efficiency and the results are outlined in Figure 3. The copper (II) ion removal efficiency was only 11.7% and the yield of melted water was 26.3% when the hydration time was 0.5 h. The copper (II) ion removal efficiency and the yield of melted water rapidly rises as the hydration time rises. The copper (II) ion removal efficiency reached 75.8% when hydration time was 4 h, and the corresponding yield of melted water was 70.6%. However, both copper (II) ion removal efficiency and the yield of melted water increased slowly after 4 h and copper removal efficiency even started to decreases lightly. These results indicate that more heavy metal ions could have become trapped between, or adsorbed onto, the surface of the hydrate crystallites when the hydration time continuously increased. Therefore, 4 h was chosen for the following experiments.

### 2.3. Effect of Copper (II) Ions Concentration on Removal Efficiency

**Figure 4:**
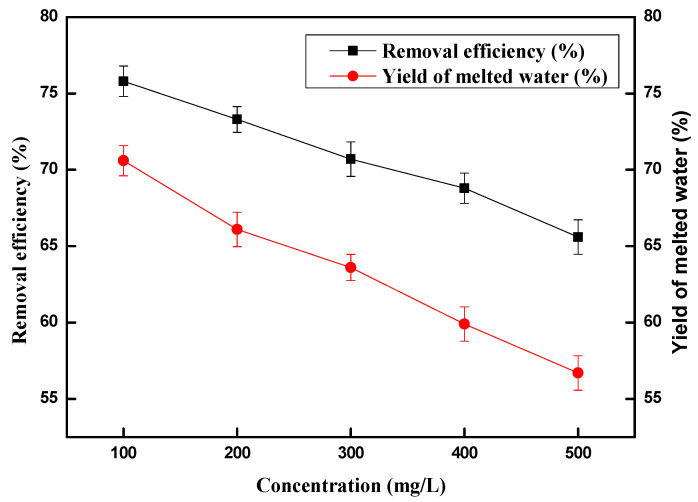
Effect of copper (II) ion concentration on removal efficiency and the yield of melted water.

The copper (II) ion concentration of an aqueous solution is an important parameter in the process of removal. As shown in Figure 4, the removal efficiency toward copper (II) ions was 75.8% and the corresponding yield of melted water was 70.6% when the model solution copper (II) ions concentrations were 100 mg/L. It can be seen that the removal efficiencies toward copper (II) ions and the corresponding yield of melted water keeps decreasing with an increase in initial concentrations. When copper (II) ion concentrations increased to 200 mg/L, 300 mg/L, 400 mg/L and 500 mg/L, the copper (II) ion removal efficiency decreased to 73.3%, 70.7%, 68.8%, and 65.6%, respectively. The yield of melted water then decreased to 66.1%, 63.6%, 59.9%, and 56.7%, respectively. To our delight, the copper (II) ion removal efficiencies and the corresponding yield of melted water obtained were 65.6% and 56.7% respectively, even with an initial copper concentration of 500 mg/L. In consideration of copper (II) ion removal efficiency and yield of melted water, we chose the model solution copper (II) ions concentration of 100 mg/L for the following experiments.

### 2.4. Effect of Stirring Speed on the Copper (II) Ions Removal Efficiency

**Figure 5:**
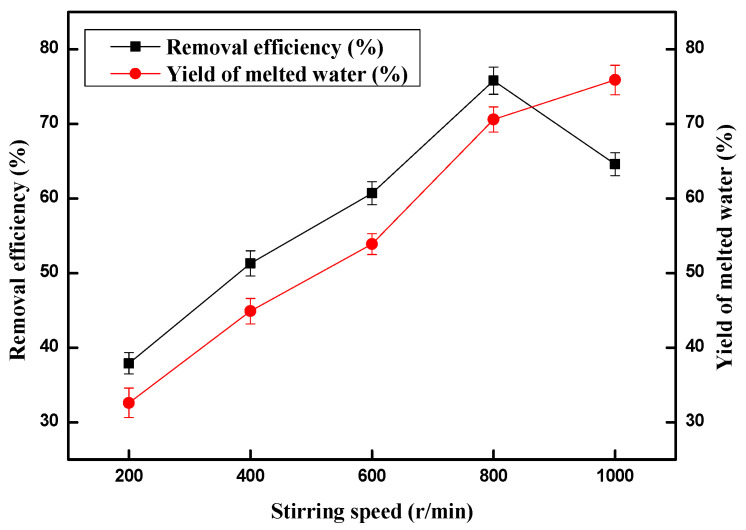
Effect of stirring speed on the removal of copper (II) ions and the yield of melted water.

On the basis of the screening of the above conditions, we further explore the effect of stirring speed on copper (II) ion removal efficiency. Stirring speed ranged from 200 r/min to 1000 r/min. The results are outlined in Figure 5. Within a certain stirring speed range, the increase of stirring speed increased copper (II) ion removal efficiency and the yield of melted water. Although copper (II) ion removal efficiency was only 37.9% with a stirring speed of 200 r/min, copper (II) ion removal efficiency increased to 51.3%, 60.7%, and 75.8% respectively when the stirring speed increased to 400 r/min, 600 r/min and 800 r/min. Unfortunately, 64.6% copper (II) ion removal efficiency was obtained while the corresponding yield of melted water obtained was 75.9% when the stirring speed increased to 1000 r/min. The reason for this is that higher stirring speed contributes to an increase in the volume of copper (II) ions from the solution that are adsorbed to the methane hydrate surface. Thus, the optimal stirring speed was 800 r/min.

## 3. Materials and Methods

### 3.1. Materials and Apparatus

**Figure 6:**
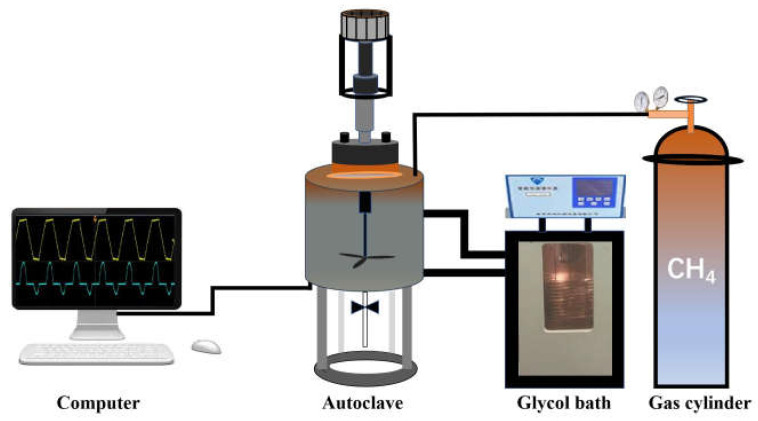
Schematic diagram of apparatus for copper (II) ions removal.

Unless otherwise stated, all chemicals and reagents were commercially available in analytical grade without further purification. Copper (II) sulfate of analytical grade was purchased from Shanghai Macklin Chemical Co. Ltd. and distilled water was produced in our laboratory. The weight of copper (II) sulfate was measured by an electronic balance with an accuracy of 0.1 mg. Inductively Coupled Plasma (ICP, Optima 8300) was used to measure the concentration of copper (II) sulfate solution. All experiments have been conducted in an autoclave with a stirrer. The experimental flow diagram is illustrated in Figure 6. The apparatus mainly consists of an autoclave, a stirrer with an adjustable rotation speed, a glycol bath with temperature control system, and a system for data acquisition and collection. A stirrer was inserted into the autoclave and the stirring speed can be controlled from 0 r/min to 1200 r/min. A thermocouple with an accuracy of ±0.1 °C was inserted from the bottom of the autoclave to monitor the temperature of experimental solutions. The temperature of experimental solutions could be controlled from −20 °C to 50 °C by a glycol bath. The temperature data can be collected by the data acquired and collected system.

### 3.2. Experimental Procedure

According to our previous work [39,40], the autoclave and the relative connections are cleaned three times, and then the prepared copper (II) sulfate solution was injected into the autoclave. Then, the glycol bath was set to the experimental temperature, and maintained for 30 min. Methane was injected into the autoclave at a certain temperature. The stirrer was started and adjusted to the required speed to form methane hydrate. After a certain time, the solution that was not intended to form methane hydrate was vented out and collected with a beaker on a balance to be weighed. When all solutions were vented into the beaker, the valve was closed to leave methane hydrate in the autoclave. Then, the temperature was set to 20 °C to dissociate methane hydrate completely and the dissociated solution was obtained. The dissociated solution was vented into another baker and then weighed by a balance. The vented solution was collected for ion measurement by Inductively Coupled Plasma (ICP). Unless otherwise stated, all the experiments were repeated two times and all experimental data are the average of two repetitions. The difference between the two repetitions is less than 1.0 wt%.

### 3.3. Data Analysis

The copper (II) ions removal efficiency is calculated using the following equation.

*R* = (*W*_0_ − *W*_1_)/*W*_0_ × 100% (1)

where *R* is the copper (II) sulfate removal efficiency, *W*_0_ is initial copper (II) sulfate concentration in solution, and *W*_1 *s*_ is copper (II) sulfate concentration in dissociated methane hydrate.

The yield of melted water is calculated using the following equation.

Yield of melted water = *V_w_*/*V_t s_* × 100% (2)

where *V_w_* is the volume of melted water obtained from methane hydrate and *V_t_* is the total volume of the initial solution.

## 4. Conclusions

In summary, we have reported an efficient and novel heavy metal copper (II) ions removal method for waste water purification via a methane hydrate-based technology. Ttemperature, hydration time, initial copper (II) ions concentration, and stirring rate played an important role in the removal efficiency and the yield of the melted water. Optimal conditions for copper (II) ion removal were: temperature −2 °C, hydrate-time 4 h, copper (II) ions concentration 100 mg/L and stirring speed 800 r/min. The removal efficiency and the according yield of melted water were 75.8% and 70.6%, respectively. Under our conditions, the initial concentration of copper (II) ions in the aqueous solution could be between 100 and 500 mg/L. This report offers a reference for a gas hydrate method for the separation of heavy metals from wastewater and demonstrated that hydrate-based technology is a potentially useful method in separation application fields.

## Data Availability

Not applicable.

## Sample Availability

Samples of the compounds are available from the authors.
